# Relationship between Internet addiction and body mass index and the predictive role of emotion dysregulation

**DOI:** 10.3389/fpsyg.2023.1305828

**Published:** 2024-01-08

**Authors:** Morteza Azizi, Behrouz Abbasi, Hajar Aghaei

**Affiliations:** ^1^Department of Psychology, Sarab Branch, Islamic Azad University, Sarab, Iran; ^2^Azerbaijan Charkhe Niloofari Higher Education Institute, Tabriz, Iran; ^3^Department of Psychology, Islamic Azad University, Tabriz Branch, Tabriz, Iran

**Keywords:** Internet, obesity, emotion dysregulation, addiction, Internet addiction

## Abstract

In recent years, the widespread use of the Internet has led to increasing concerns about problematic behaviors related to excessive Internet use and their potential consequences. This study aimed to investigate the relationship between Internet addiction (IA), body mass index (BMI), and emotion dysregulation (ED). Specifically, the study aimed to determine if IA significantly predicts obesity and if both Internet addiction and obesity can be significantly predicted by ED. 367 school-attending adolescents (M_age_ = 13.35; *SD* = 0.82; 49% girls) in Tekab participated in the study. Participants completed the Difficulties in Emotion Regulation Scale (DERS) and the Internet Addiction Test (IAT), while BMI was calculated using self-reported data to assess their obesity levels. The results indicated that ED significantly predicted both IA and increased BMI levels (*p* < 0.001). Furthermore, IA also significantly predicted elevated BMI levels (*p* < 0.001). Our findings showed that ED significantly predicted both higher IA and BMI values, while IA also significantly predicted elevated BMI levels These results have important implications for treatment. To address excessive Internet use or overeating behavior in individuals with either condition, it may be necessary to target the underlying emotional dysregulation that contributes to the problem.

## Introduction

Over the recent decades, technological developments have fundamentally shaped the role of the Internet in our lives (Elhami Athar and Azamian Jazi, 2021). Today, the Internet plays a crucial role in education, work, and leisure activities for many people (Maree, 2017). However, this widespread use has also led to growing concerns about problematic Internet behaviors and related conditions. In this regard, Young (2004) indicated that an individual could be diagnosed with Internet addiction (IA) if he/she meets at least five of the eight diagnostic criteria during a 6-month period. Likewise, a novel diagnostic condition, namely “Internet Gaming Disorder (IGD),” has been suggested to be included as a condition for further study in the latest edition of the *Diagnostic and Statistical Manual of Mental Disorders* (American Psychiatric Association, 2013).

Overall, excessive use of the Internet has adverse effects on mental health (Griffiths, 2019; Longobardi et al., 2020). IA is significantly associated with low life satisfaction (Satici and Uysal, 2015), poor academic performance (Al-Yafi et al., 2018), depression (Satici, 2019), social anxiety (Atroszko et al., 2018), poor work performance, insomnia, and suicide ideation or commitment (Brailovskaia et al., 2019, 2020). Meanwhile, one of the most significant consequences of excessive Internet use is the alteration of body fat distribution, leading to an increase in body weight and obesity (Barrense-Dias et al., 2016; Park and Lee, 2017). Obesity could directly or indirectly lead to various chronic diseases and cause high costs both individually and socially; thus, it has become a prioritized matter within the scope of protecting and improving health. About 80% of the world’s adolescent population does not make adequate physical activity (Aghasi et al., 2019). Prolonged sitting during Internet use may contribute to sedentary behavior among adolescents. Furthermore, the addition of unhealthy eating habits such as snacking to prolonged screen time increases the risk of obesity. Moreover, the COVID-19 pandemic has led to increased Internet use and the lockdowns and other measures taken to combat the pandemic may have long-term consequences on the obesity epidemic (for a review see Aghasi et al., 2019).

Meanwhile, IA and obesity could be associated with emotion regulation difficulties. Emotion regulation (ER) refers to the ability to adjust emotional arousal and accomplish goal-directed behaviors regardless of emotional state; deficits in ER or emotion dysregulation (ED) lead to difficulties in monitoring, evaluating, or adjusting emotional reactions (Gratz and Roemer, 2004; Gross, 2013). Prior studies have suggested the etiological role of ED on obesity (Aparicio et al., 2016; Andrei et al., 2018). More specifically, a meta-analysis showed that individuals with obesity had difficulties in identifying feelings, emotional awareness, and using appropriate emotional regulation strategies when compared with a control group (Fernandes et al., 2018). Additionally, individuals with ED might utilize eating as a coping mechanism in dealing with negative emotions. In other words, they may engage in emotional eating or using food to cope with negative emotions, which is directly associated with weight gain and obesity (Leehr et al., 2015). Also, individuals with ED might have problems in stress management, which can lead to overeating and weight gain (Thompson, 2019). In the same vein, studies have shown the relationships between ED and IA (Spada and Marino, 2017; Cimino and Cerniglia, 2018). As Internet use helps one avoid reality and be distracted from stress, it could function as a dysfunctional emotional regulation strategy (Mo et al., 2018). Internet use may provide relief from problematic emotions in the short term but when used as an emotional strategy, it is negatively reinforced and may lead to IA (Mo et al., 2018). In addition, Yilmaz Kafali et al. (2021) showed that the IA significantly mediated the association between ED and BMI, while ED and IA significantly predicted obesity (Yilmaz Kafali et al., 2021).

Still, the relationship between the ED and IA has been debated. For instance, Donald et al. (2020) did not find evidence that emotion regulation difficulties preceded the development of compulsive Internet use and suggested that teaching general emotion regulation skills may not be as effective in reducing compulsive Internet use. Likewise, Bélanger et al. (2011) did not find significant correlations between Internet use and being overweight. Therefore, more studies are needed to examine how these variables are related to each other. All these taken into account, the present study was conducted as an attempt to fill the gap in the literature. In this vein, we first examine if Internet use predicts increased BMI levels. Then, we explore whether ED significantly predicts IA and increased BMI values.

## Methods

### Participants and procedure

Participants were 367 school-attending youth aged 10–14 years (M_age_ = 13.35; *SD* = 0.82; 49% girls) old who were recruited from schools in Tekab between March 2021 to June 2021. Before gathering data, the students and their teachers were explained about the aims and process of the study. They were then informed about the data’s confidentiality and that it would only be used for the present study. After providing their informed consent, participants were asked to fill out the measures, while they could ask questions from the data gatherer if they needed. The participants completed the questionnaires in their classroom during a one-hour session under the supervision of a specially trained research assistant (master-level student). This study was approved by the ethics committee of the Islamic Azad University, Sarab Branch. Also, approval was provided by the Iran Ministry of Education and the boards of each school.

### Measures

#### Internet addiction test

The Internet addiction test (IAT) was developed by Young (1998) and is the first validated and reliable measure for assessing the addictive use of the Internet. IAT has 20 items and measures psychological dependence, compulsive use, withdrawal, and related problems of school, sleep, family, and time management as a result of addictive Internet use. Items are rated on a 5-point Likert-type scale ranging from 1 (*rarely*) to 5 (*always*). The minimum obtainable score on the IAT is 20, and the maximum is 100, with higher scores indicating a greater level of Internet addiction. Persian version of the IAT yielded acceptable psychometric properties with Iranian samples (Elhami Athar and Azamian Jazi, 2021). The Cronbach’s alpha for the IAT score in this study was 0.92.

#### Difficulties in emotion regulation scale

The difficulties in emotion regulation scale (DERS; Gratz and Roemer, 2004) is a 36-item self-report questionnaire that assesses emotion dysregulation. The DERS items load on six subscales, including Lack of Emotional Awareness (6 items), Lack of Emotional Clarity (5 items), Difficulties Controlling Impulsive Behaviors When Distressed (6 items), Difficulties Engaging in Goal-Directed Behavior When Distressed (5 items), Nonacceptance of Negative Emotional Responses (6 items), and Limited Access to Effective ER Strategies (8 items). Participants rate items on a 5-point scale ranging from 1 (*almost never*) to 5 (*almost always*). A total score is obtained by summing all items. The internal consistency and validity of the Persian version of DERS were supported with the Iranian sample in previous studies (Besharat and Bazzazian, 2013; Vafa et al., 2021). The Cronbach’s alpha for the DERS score in this study was 0.95.

#### Body mass index

We calculated the Body mass index (BMI) scores of the participants according to the following formula: BMI = Weight (kg)/Height (Meter^2^) based on the self-reported data.

### Data analyses

We first explored descriptive statistics for study variables. Then we calculated zero-order Pearson correlations between the variables, which were interpreted as ≤0.30 = small; 0.30–0.50 = medium; and 0.50 ≤ strong effect sizes (Cohen, 2013). Next, three separate simple linear regression analyses were conducted. In the first regression, we examined if ED predicts IA. For the second and third regressions, the dependent variable was BMI, while the predicting variables were IA and ED, respectively. To assess multicollinearity in each regression model, Variance Inflation Factors (VIF) were calculated. The results showed that all VIF values in each model were within this acceptable range of (<5; Akinwande et al., 2015). Additionally, the normality of the distribution for variables was tested using the Kolmogorov–Smirnov test, and the results supported the normality of the data (*p* > 0.05). For hypothesis testing, we considered *p* < 0.05 as indicating statistically significant results. We used SPSS 20 software for data entry and statistical analyses.

## Results

Descriptive statistics of the study variables are presented in Table 1. We first conducted zero-order correlations between study variables (Table 1). The results showed that higher IA and ED scores were significantly and positively correlated with elevated BMI values (*r’*s = 0.32 and 0.35, respectively). Likewise, IA demonstrated a significant positive correlation with ED (*r* = 0.39; Table 1). We then conducted three separate simple linear regression analyses. In the first regression analysis, we were interested in examining if ED predicts IA. Our results indicated that ED explained 29% of the variance in IA, *R^2^* = 0.29, *F*(1, 365) = 18.83, *p* < 0.001. Next, in a separate regression analysis, we examined if IA predicts higher BMI levels, with the results showing that the model explained 0.04% of the variance in the outcome variable *R^2^* = 0.04, *F*(1, 365) = 7.73, *p* < 0.001. Finally, we tested whether ED predicts elevated BMI levels. Our findings supported this predictive relationship, indicating that ED explained 0.027% of the variance in BMI, *R^2^* = 0.027, *F*(1, 365) = 10.13, *p* < 0.001 (see Table 2).

**Table 1:** Descriptive statistics and bivariate correlations between study variables (*n* = 367).

		1	2	3	Mean (*SD*)	Skewness	Kurtosis	*α*
1	DERS	-	-	-	101.069 (21.06)	−0.16	−0.48	0.95
2	Internet addiction test	0.39^**^	-	-	43.03 (16.38)	0.41	−0.52	0.92
3	Body mass index	0.35^**^	0.32^**^	-	20.9 (3.60)	0.91	−0.75	-

DERS, difficulties in emotion regulation Scale; SD, standard deviation; α, Cronbach’s Alpha. ** = 0.001.

**Table 2:** Results of Simple Linear Regressions (n = 367).

			Unstandardized coefficients		Standardized coefficients				
	Predictor	Outcome variable	*B*	*SE*	*β*	*p*	*R^2^*	*F*	*p*
Regression 1	Emotion dysregulation	Internet addiction	0.46	0.12	0.32	0.001	0.29	18.83	0.001
Regression 2	Internet addiction	Body mass index	0.20	0.11	0.12	0.001	0.04	7.73	0.001
Regression 3	Emotion dysregulation	Body mass index	0.22	0.12	0.10	0.001	0.03	10.13	0.001

## Discussion

In the current study, we aimed to examine whether IA predicts obesity and if these two variables are significantly predicted by ED. We first examined correlations between these variables, and our results showed that higher IA and ED were significantly and positively correlated with elevated BMI levels. Likewise, IA demonstrated a significant positive correlation with ED. Furthermore, results from regression analyses indicated that IA significantly predicted higher BMI values, while these two variables were significantly predicted by ED.

With respect to the relationship between Internet use and the odds of being overweight and obesity (i.e., elevated BMI levels), previous studies have yielded inconsistent results (for a review see Aghasi et al., 2019). For example, Bélanger et al. (2011) failed to find a significant association between Internet use and obesity among girls. On the other hand, consistent with our results, several studies indicated that adolescents with IA disorder are more susceptible to being overweight or obese (Kautiainen et al., 2005; Berkey et al., 2008; Vandelanotte et al., 2009; Matusitz and McCormick, 2012; Peltzer et al., 2014). Individuals who spend more time on the Internet tend to engage in sedentary behavior, such as sitting for long periods of time which is associated with decreased physical activity and decreased energy expenditure that can lead to weight gain and decreased physical activity (Dunstan et al., 2010). Additionally, Internet use may also be associated with unhealthy dietary habits, such as snacking, drinking, and consuming high-calorie foods while sitting in front of a computer. Such a lifestyle could be a significant risk factor for overweight and obesity (Vandelanotte et al., 2009; Aghasi et al., 2019; Schaan et al., 2019). Excessive Internet use is also associated with disruptions in sleep patterns and social isolation, both of which are associated with an increased risk of obesity (Berkman et al., 2000; Holt-Lunstad et al., 2010).

In addition, our findings showed that ED significantly predicted IA. Prior studies indicated that disturbed emotion regulation abilities anticipated more elevated levels of IA (Yildiz, 2017; Pettorruso et al., 2020). Some studies have found that individuals with higher levels of ED are more likely to engage in excessive Internet use as a means of coping with negative emotions and stress; in other words, individuals with ED problems may use the Internet as a form of emotional escape. When they are feeling overwhelmed, anxious, or sad, they may engage in excessive Internet use as a way to distract themselves from their negative emotions and to feel a temporary sense of relief (Kuss et al., 2017; Sinha et al., 2021). Relatedly, various unfavorable life events may bring about negative emotions, and the incapacity to manage or regulate these emotions may lead to high levels of impulsivity and the development of dysfunctional behaviors such as excessive Internet use (Rogier and Velotti, 2018). Also, the Internet provides a sense of social connection to individuals suffering from ED. These individuals may have difficulty regulating their emotions in social situations, leading them to feel isolated and lonely. By using the Internet, they can connect with others and reduce feelings of loneliness and social isolation (Kuss and Griffiths, 2011; Lee and Stapinski, 2012). Finally, in line with previous studies, our results indicated that ED significantly predicted higher BMI values (Leehr et al., 2015; Rogier and Velotti, 2018). In this regard, studies suggest that eating may comprise a coping mechanism for negative emotions. Individuals who struggle with emotion regulation may be more likely to engage in emotional eating, which involves using food to cope with negative emotions. That is, negative emotions could initiate overeating (i.e., eating a large amount of food) or binge eating (i.e., eating a large amount of food with a loss of control), which are directly associated with weight gain and obesity (Leehr et al., 2015). Additionally, individuals with ED may have difficulties with stress management, which can lead to overeating and weight gain (Thompson, 2019).

The current study results should be interpreted concerning a few limitations. First, we used only self-report measures for the data gathering, so correlations between self-report measures may partly be explained by shared method variance. Relatedly, it is imperative to address potential limitations associated with the self-reported nature of height and weight data utilized in calculating BMI. The reliance on self-reported data for BMI calculations may lead to reporting bias, where participants may unintentionally misreport their height and weight due to various factors such as social desirability or personal perception. Future studies are recommended to utilize more objective measures, such as clinical assessments or anthropometric measurements, to enhance the precision and accuracy of BMI assessments. Second, the fact that we only included students from schools located in one city limits the generalization of the results. Third, this study had a cross-sectional design, which prevents us from establishing causal relationships between the variables. To better understand the dynamic relationships and potentially discover reciprocal influences within this complex interplay, longitudinal studies are necessary. Such investigations would offer a deeper understanding of the temporal aspect and provide valuable insights into the temporal sequence and possible bidirectional influences between Internet addiction, emotion dysregulation, and obesity. Fourth, our study employed a dimensional approach to IA and did not specifically examine individuals meeting the criteria for IA or those recognized as obese based on their BMI levels. Future research should consider replicating our analyses with samples specifically composed of individuals meeting the criteria for IA and exhibiting elevated BMI levels. This targeted investigation would contribute to a more nuanced understanding of the intricate interplay among these variables, enhancing the generalizability and robustness of findings across different populations.

## Implications for practice and future research

The outcomes of this study hold substantial implications for clinical practice which should be examined in future research. Specifically, when considering the practical implications of our findings, especially the influential roles of ED in IA and BMI, a clear emphasis emerges on treatments that focus on enhancing emotion regulation. Tailoring emotional wellness programs to empower individuals in effectively navigating negative emotions could present a promising approach to addressing both Internet addiction and unhealthy eating behaviors, potentially contributing to elevated BMI levels. Such interventions may play a pivotal role in adopting a comprehensive perspective, acknowledging and addressing the intricate interplay between emotional regulation, Internet use, and outcomes related to weight.

## Conclusion

The findings of the present study reveal a significant predictive relationship between IA and increased BMI, with ED playing a pivotal role in predicting elevated levels of both IA and BMI. The recognition of ED as a predictor in IA and BMI has noteworthy treatment implications. The results imply that future research should explore the effectiveness of treatment approaches that focus on mitigating underlying emotional dysregulation contributing to excessive Internet use or overeating behavior. This may entail therapeutic interventions aimed at enhancing emotional regulation skills and addressing any latent emotional or psychological issues that may be contributing to problematic behaviors. These insights underscore the potential value of a holistic treatment approach that addresses emotional well-being in individuals exhibiting symptoms of IA and elevated BMI.

## Data availability statement

The raw data supporting the conclusions of this article will be made available by the authors, without undue reservation.

## Ethics statement

The studies involving humans were approved by the Ethics Committee of the Islamic Azad University, Sarab Branch. Also, approval was provided by the Iran Ministry of Education and the boards of each school. The studies were conducted in accordance with the local legislation and institutional requirements. Written informed consent for participation in this study was provided by the participants' legal guardians/next of kin.

## Author contributions

MA: Conceptualization, Formal Analysis, Methodology, Project administration, Software, Supervision, Writing – original draft, Writing – review & editing. BA: Data curation, Formal Analysis, Methodology, Software, Writing – original draft. HA: Data curation, Methodology, Software, Writing – original draft.
